# Supplementary material for: Proximity labeling of protein complexes and cell-type-specific organellar proteomes in Arabidopsis enabled by TurboID
Source: eLife. 2019 Sep 19;8:e47864. doi: 10.7554/eLife.47864 (PMC6791687; doi:10.7554/eLife.47864)

Extended supplement – uncropped immunoblots

Figure 1B

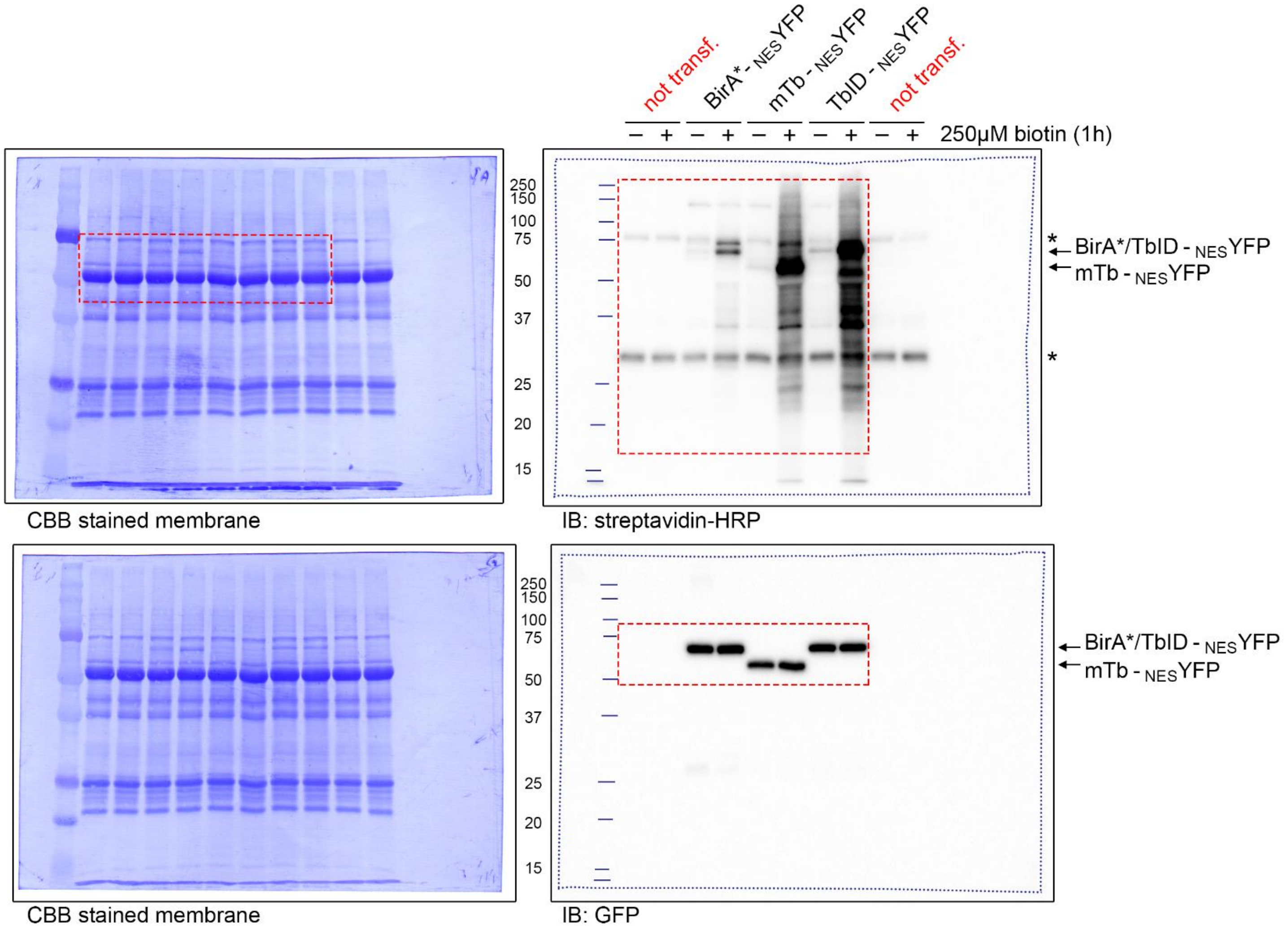

Figure 1C

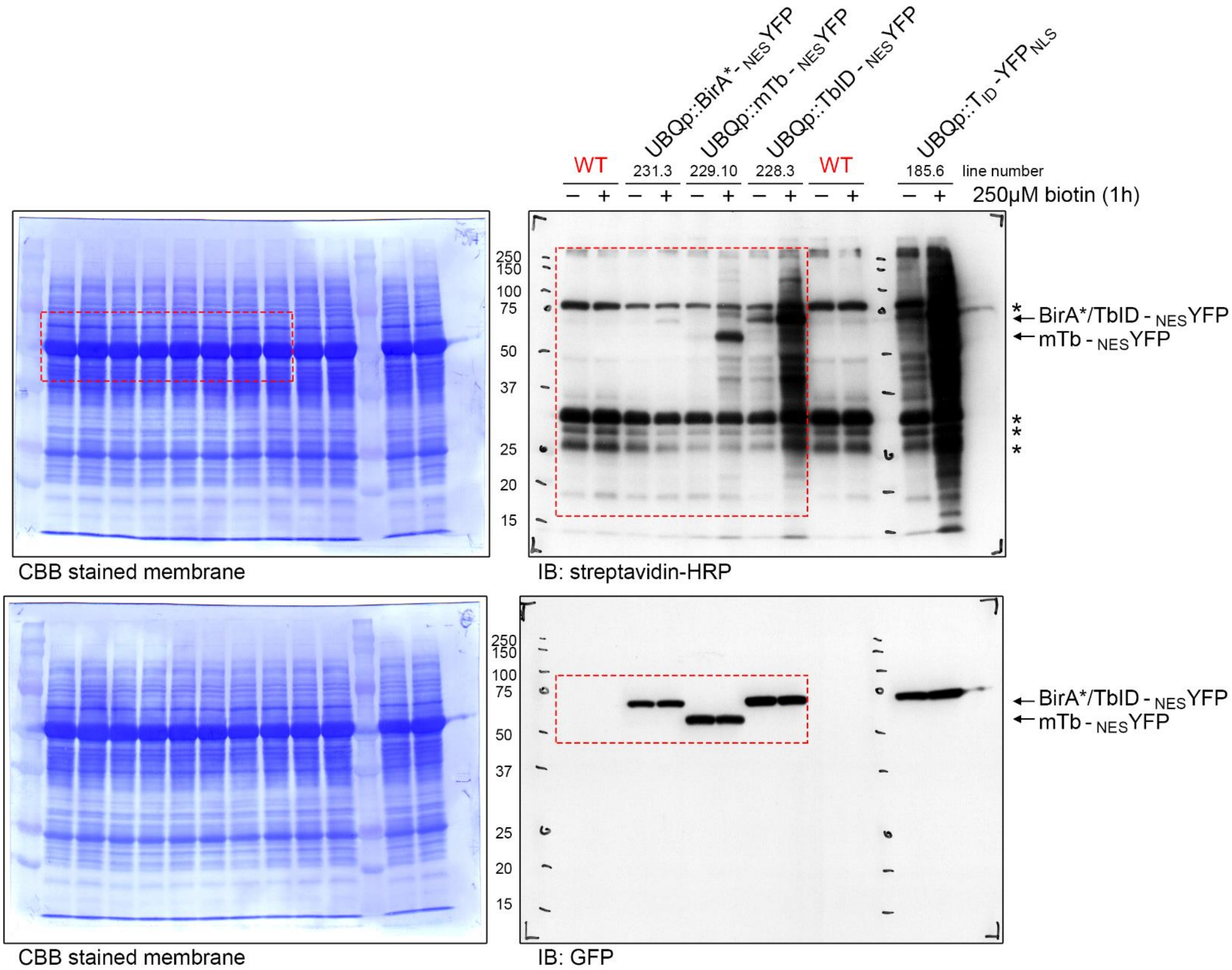
UBQp::BirA*-NESYFP
UBQp::mTb-NESYFP
UBQp::TbID-NESYFP
UBQp::TID-YFPNLS
WT
231.3
229.10
228.3
WT
185.6
line number
250µM biotin (1h)
250
150
100
75
50
37
25
20
15
BirA*/TbID-NESYFP
mTb-NESYFP
CBB stained membrane
IB: streptavidin-HRP
CBB stained membrane
IB: GFP

Figure 1 – figure supplement 1

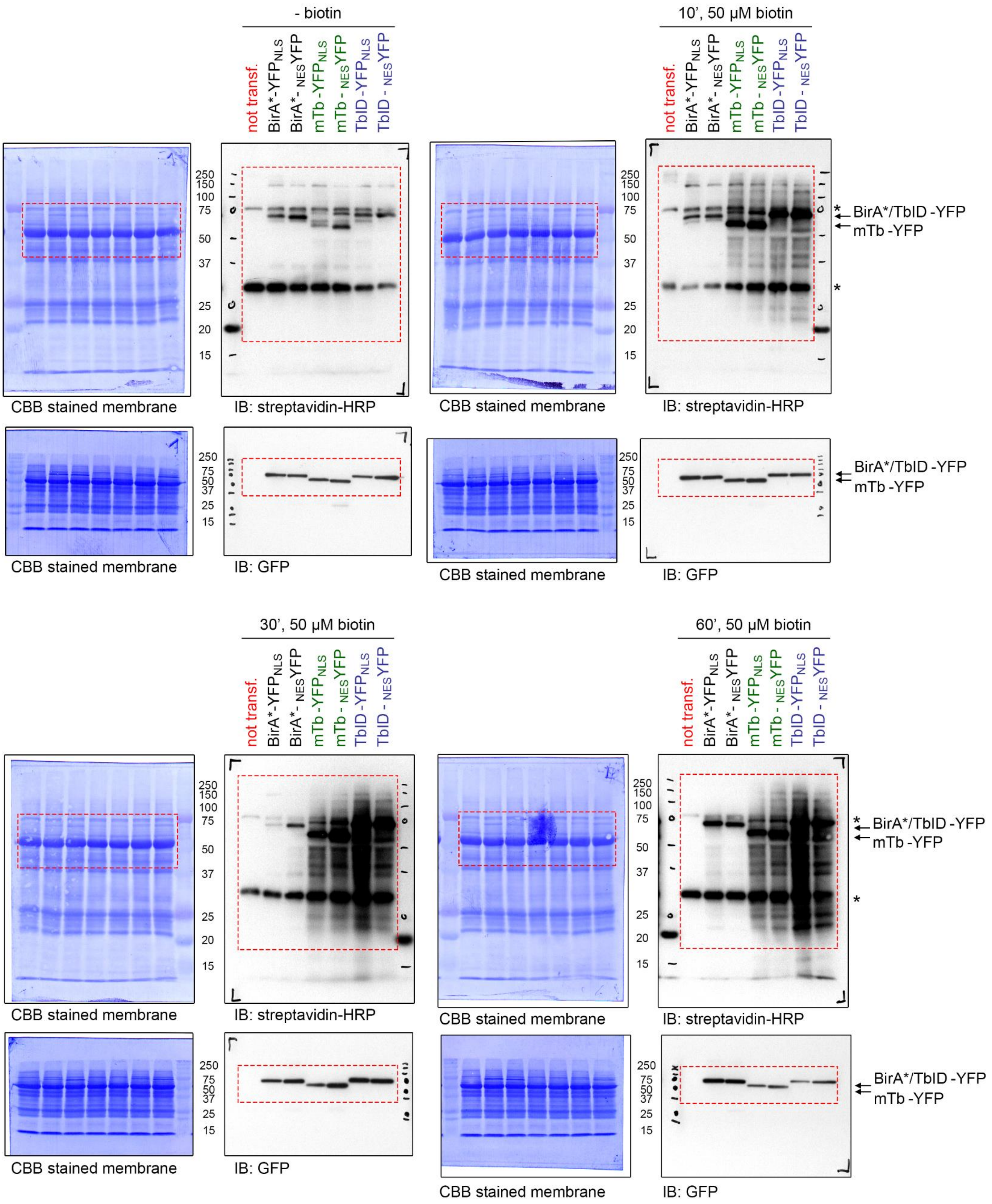

Figure 1 – figure supplement 3

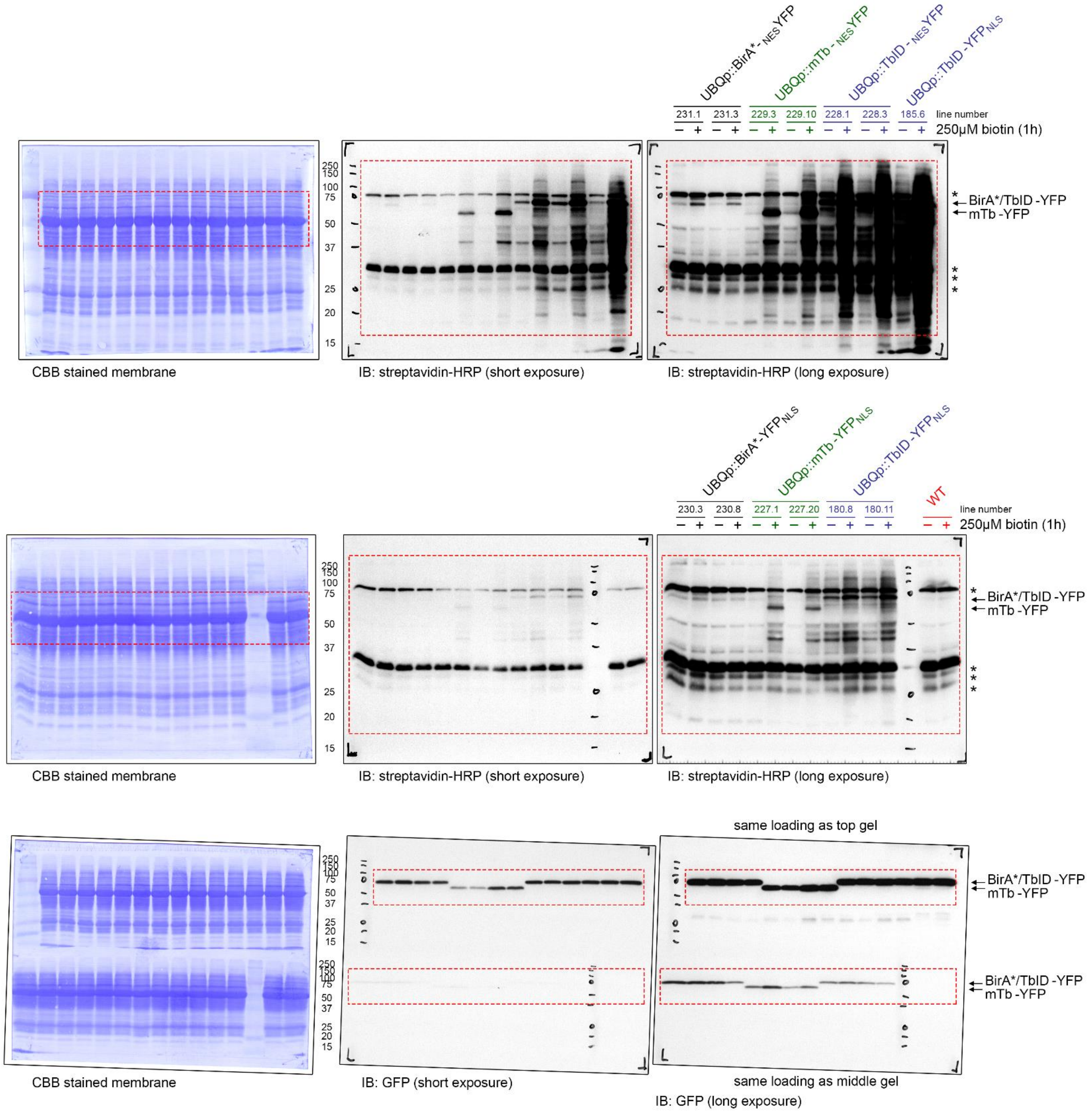

Figure 2A

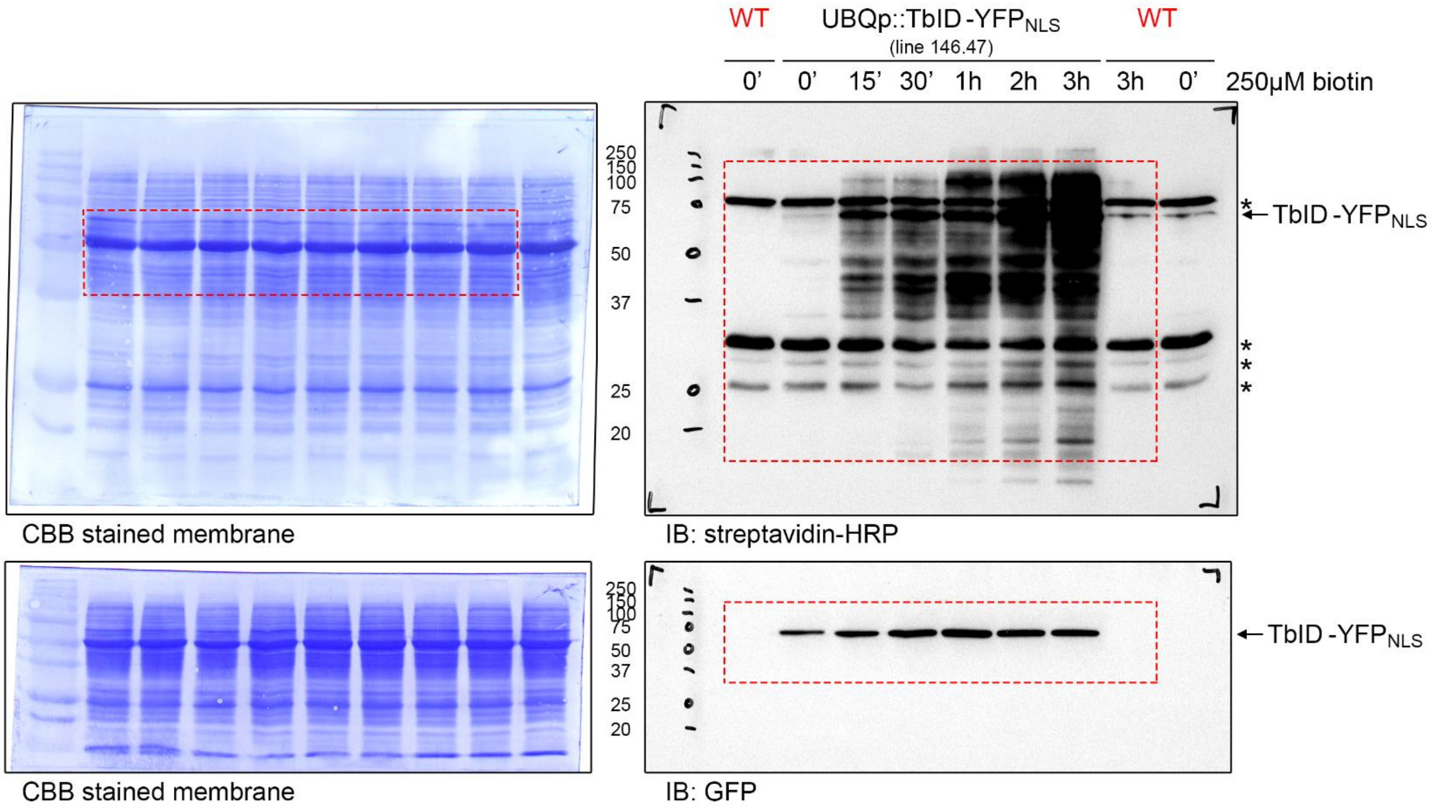
WT
UBQp::TbID-YFP NLS
(line 146.47)
WT
0’ 0’ 15’ 30’ 1h 2h 3h 3h 0’
250μM biotin
250
150
100
75
50
37
25
20
TbID-YFP NLS
CBB stained membrane
IB: streptavidin-HRP
CBB stained membrane
IB: GFP

Figure 2B

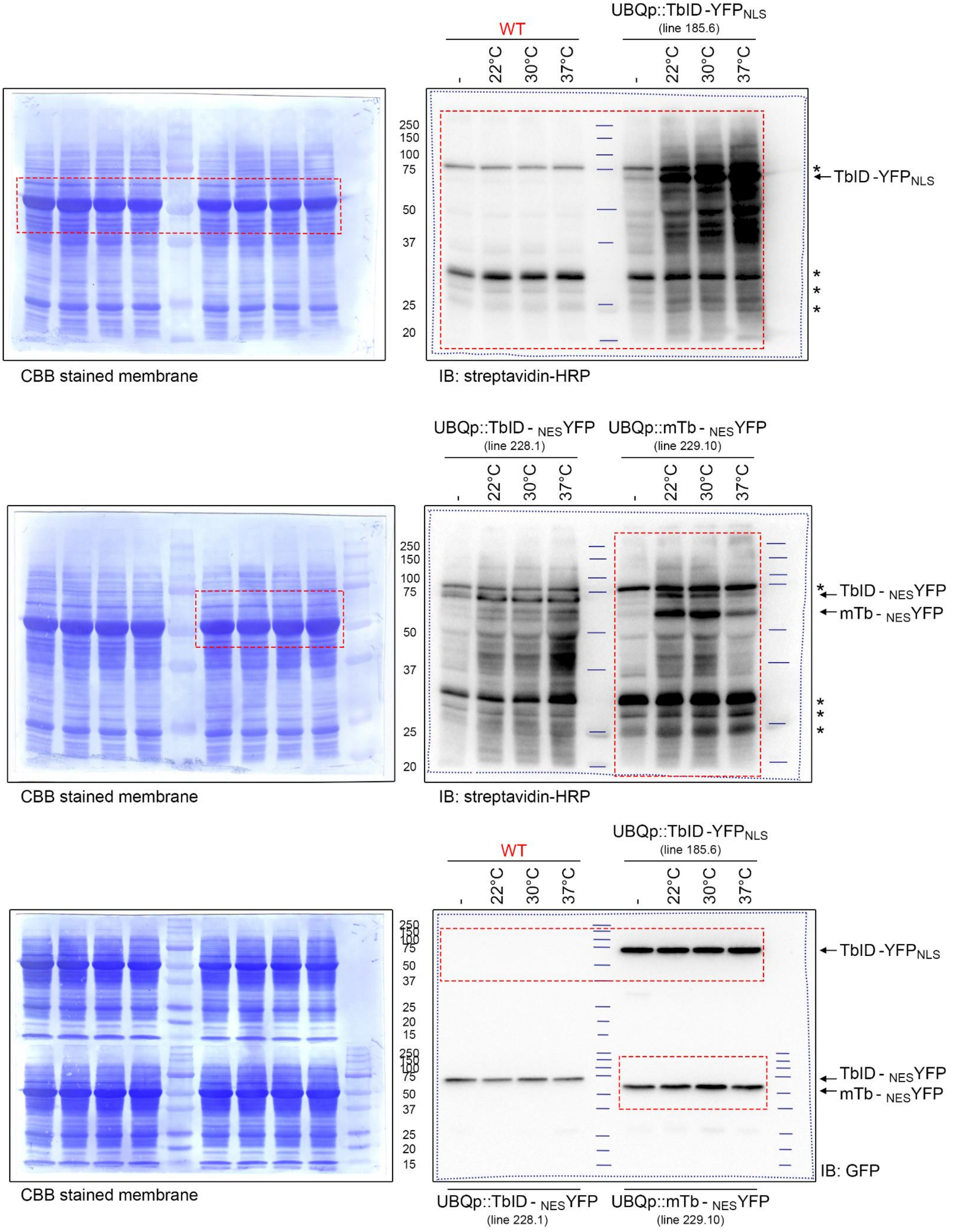
WT
UBQp::TbID-YFP_NLS
(line 185.6)
-
22°C
30°C
37°C
250
150
100
75
50
37
25
20
15
*
TbID-YFP_NLS
CBB stained membrane
IB: streptavidin-HRP
UBQp::TbID-_NES YFP
(line 228.1)
UBQp::mTb-_NES YFP
(line 229.10)
TbID-_NES YFP
mTb-_NES YFP
IB: GFP

Figure 2C and Figure 2 – figure supplement 3

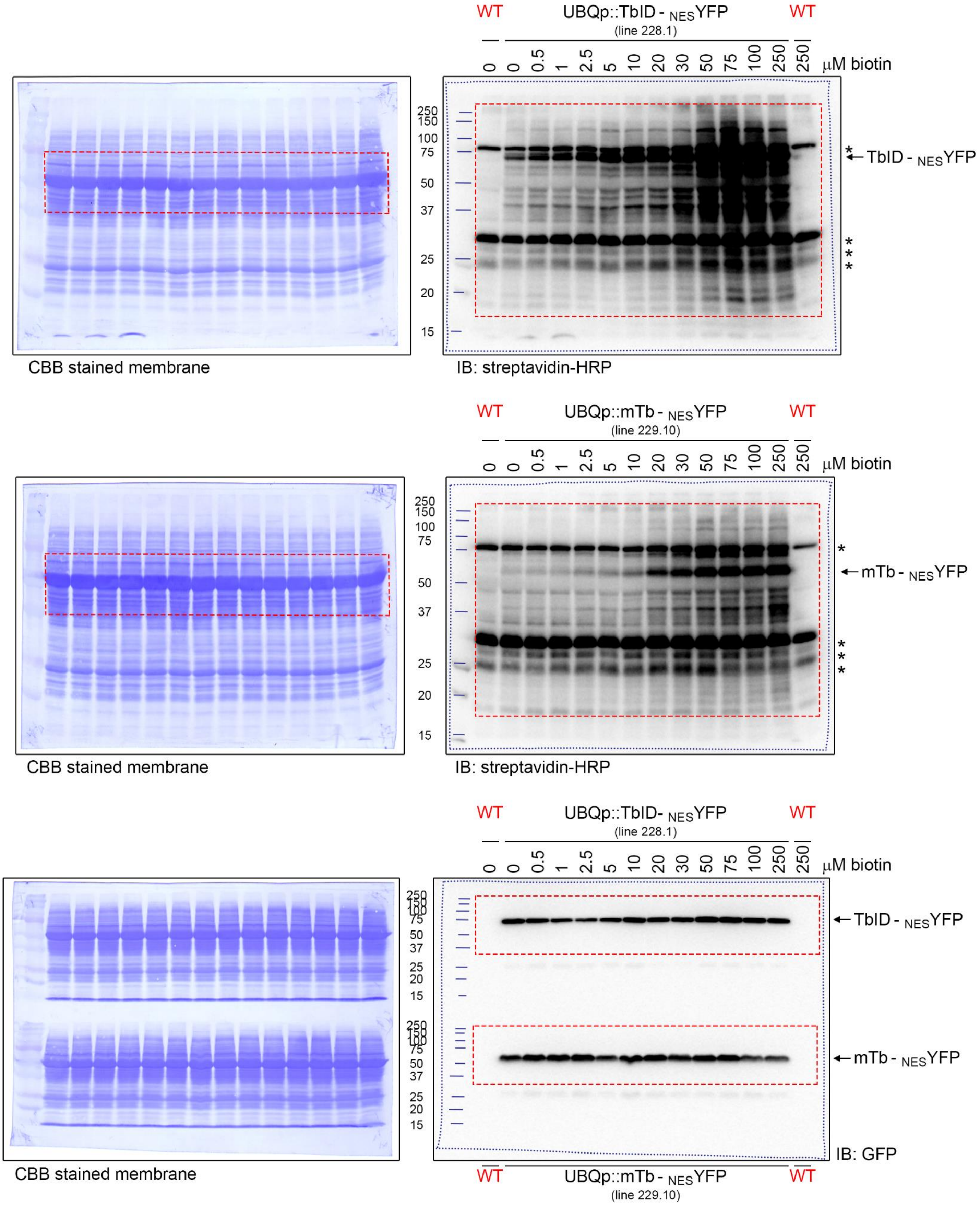

Figure 2D

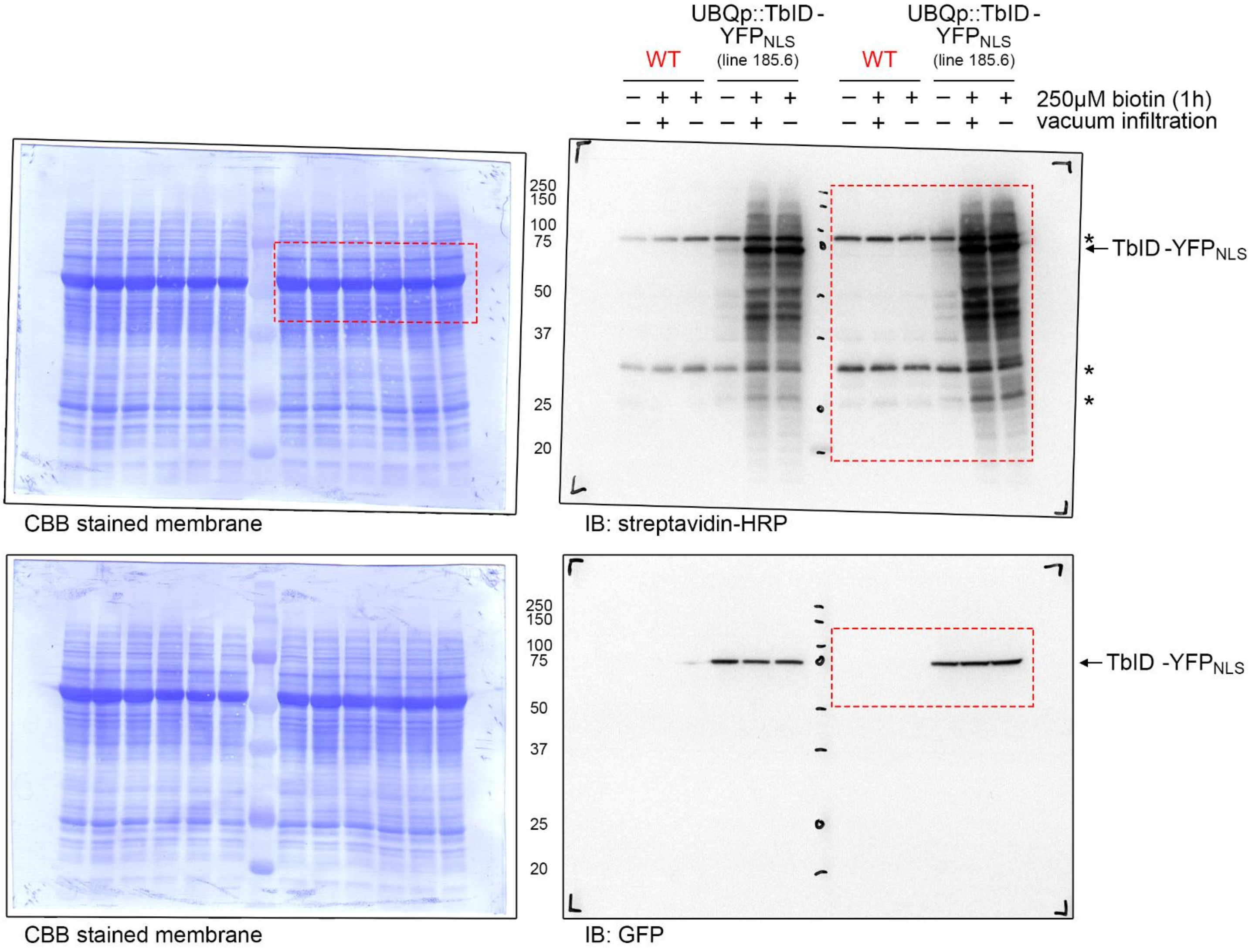

UBQp::TbID-YFP$_{NLS}$ (line 185.6)
WT
UBQp::TbID-YFP$_{NLS}$ (line 185.6)
WT
250µM biotin (1h)
vacuum infiltration
250
150
100
75
50
37
25
20
TbID-YFP$_{NLS}$
*
CBB stained membrane
IB: streptavidin-HRP
CBB stained membrane
IB: GFP

Figure 2 – figure supplement 1

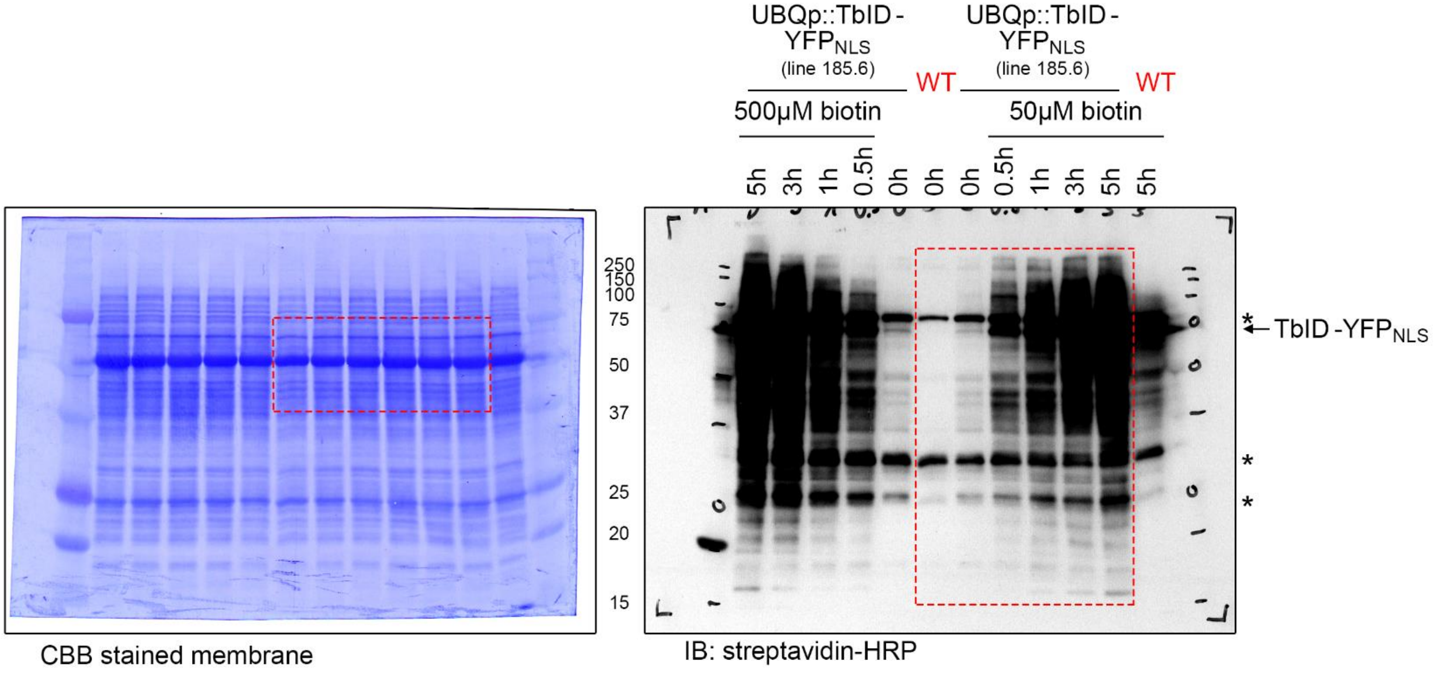

Figure 2 – figure supplement 2

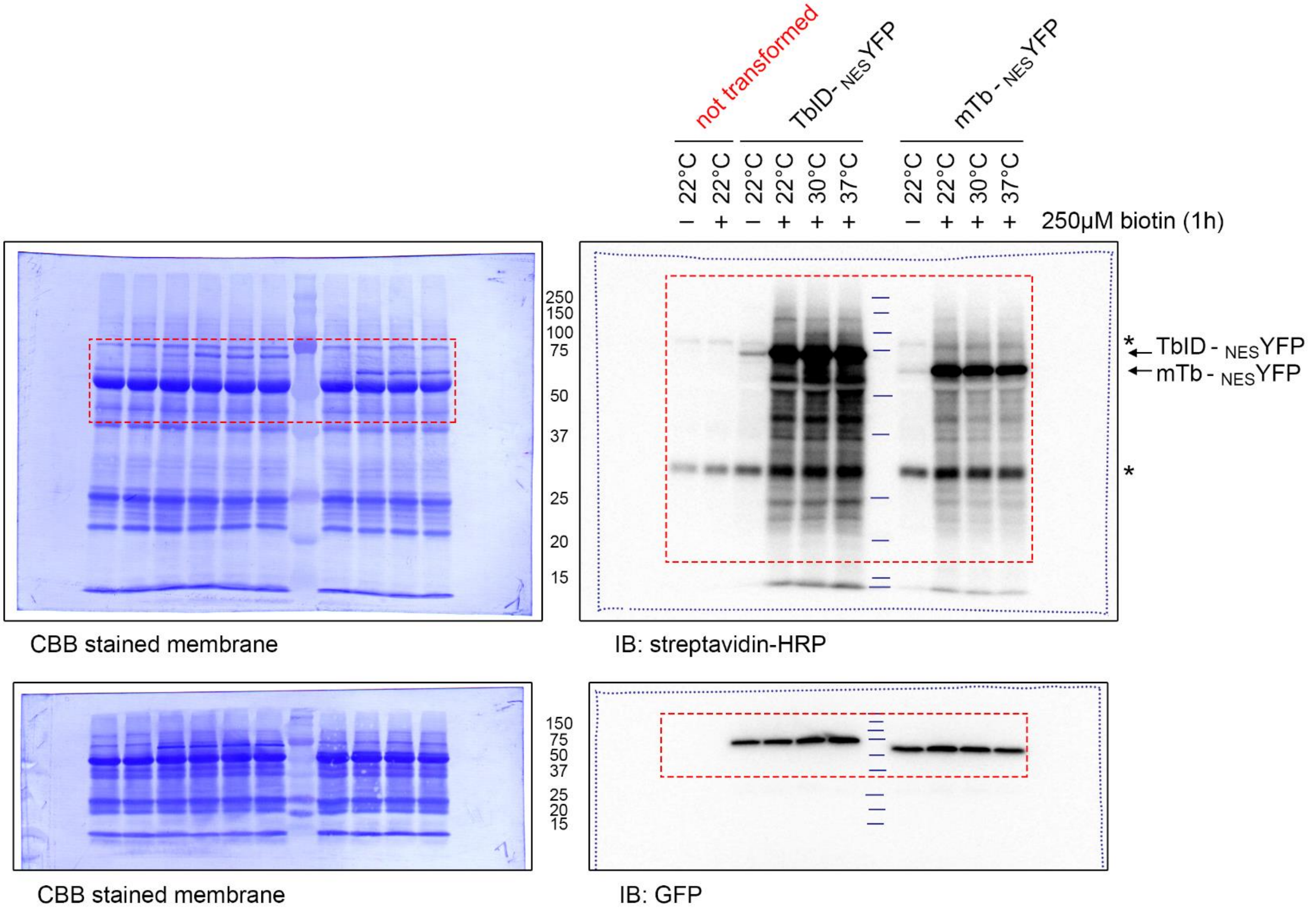

Figure 3

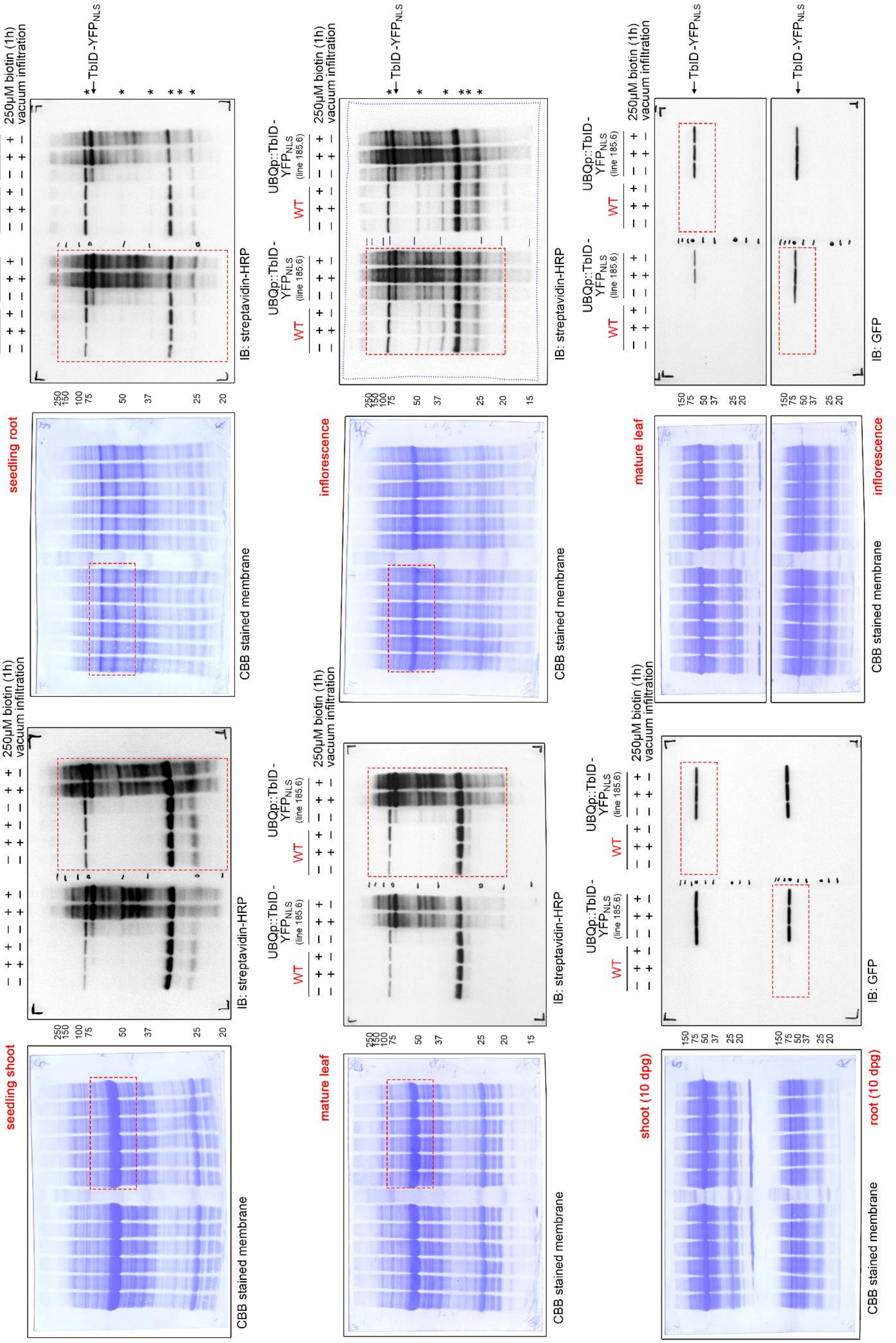

Figure supplement – uncropped immunoblots: page 10

Figure 3 – figure supplement 1

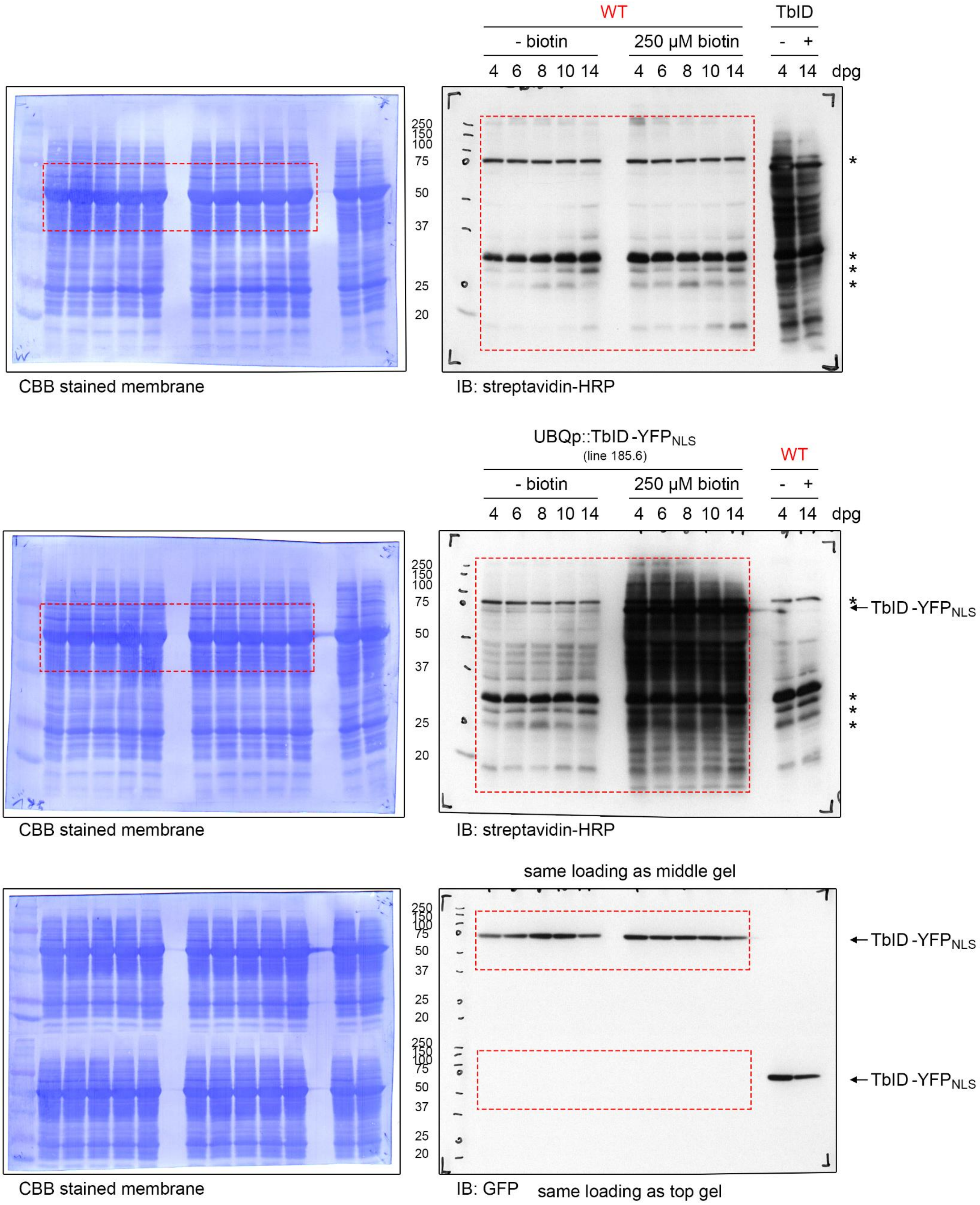

Figure 3 – figure supplement 2

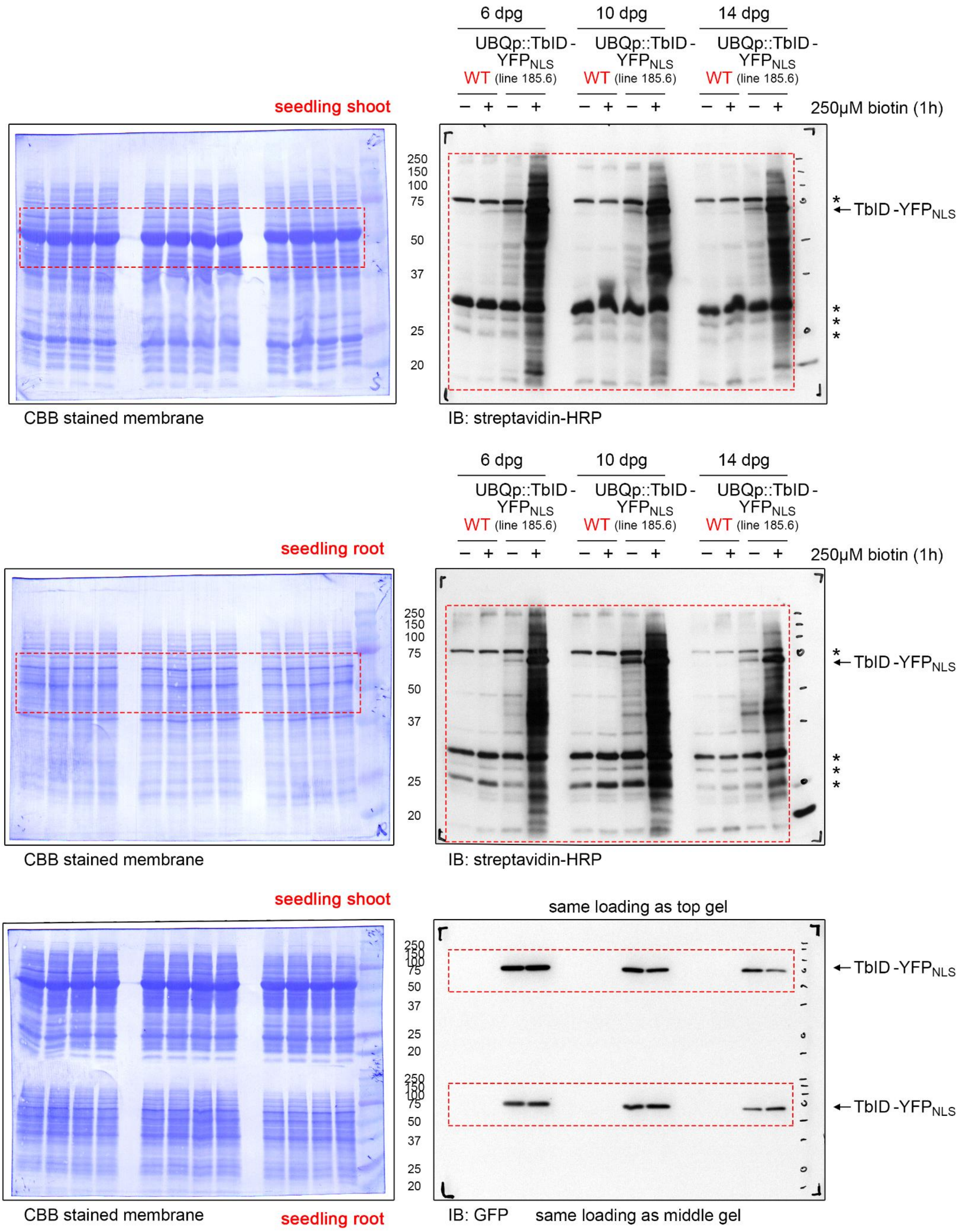

Figure 4B

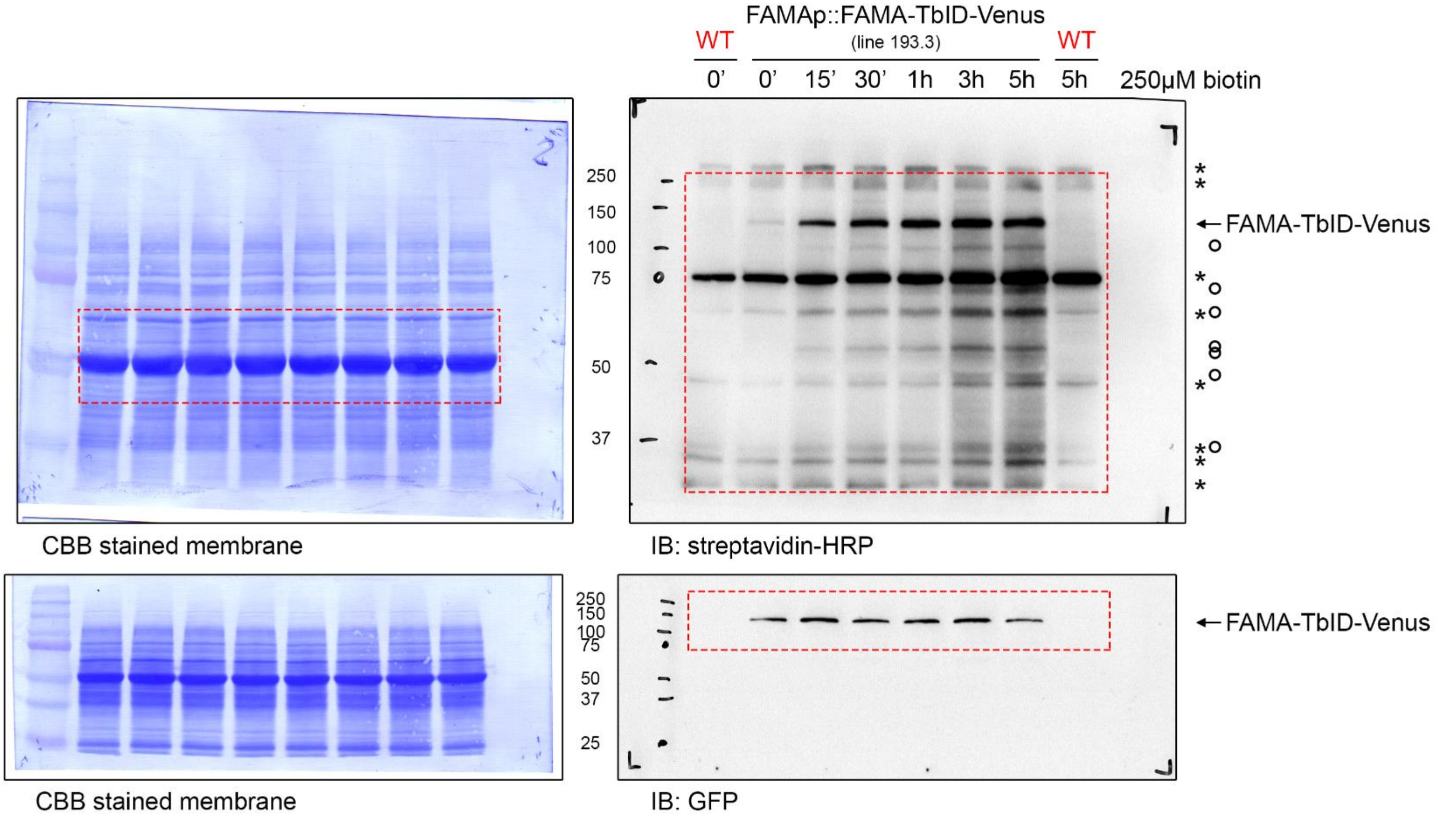
FAMAp::FAMA-TbID-Venus
(line 193.3)
WT
0'
0'
15'
30'
1h
3h
5h
WT
5h
250μM biotin
250
150
100
75
50
37
FAMA-TbID-Venus
CBB stained membrane
IB: streptavidin-HRP
250
150
100
75
50
37
25
FAMA-TbID-Venus
CBB stained membrane
IB: GFP

Figure 4 – figure supplement 3

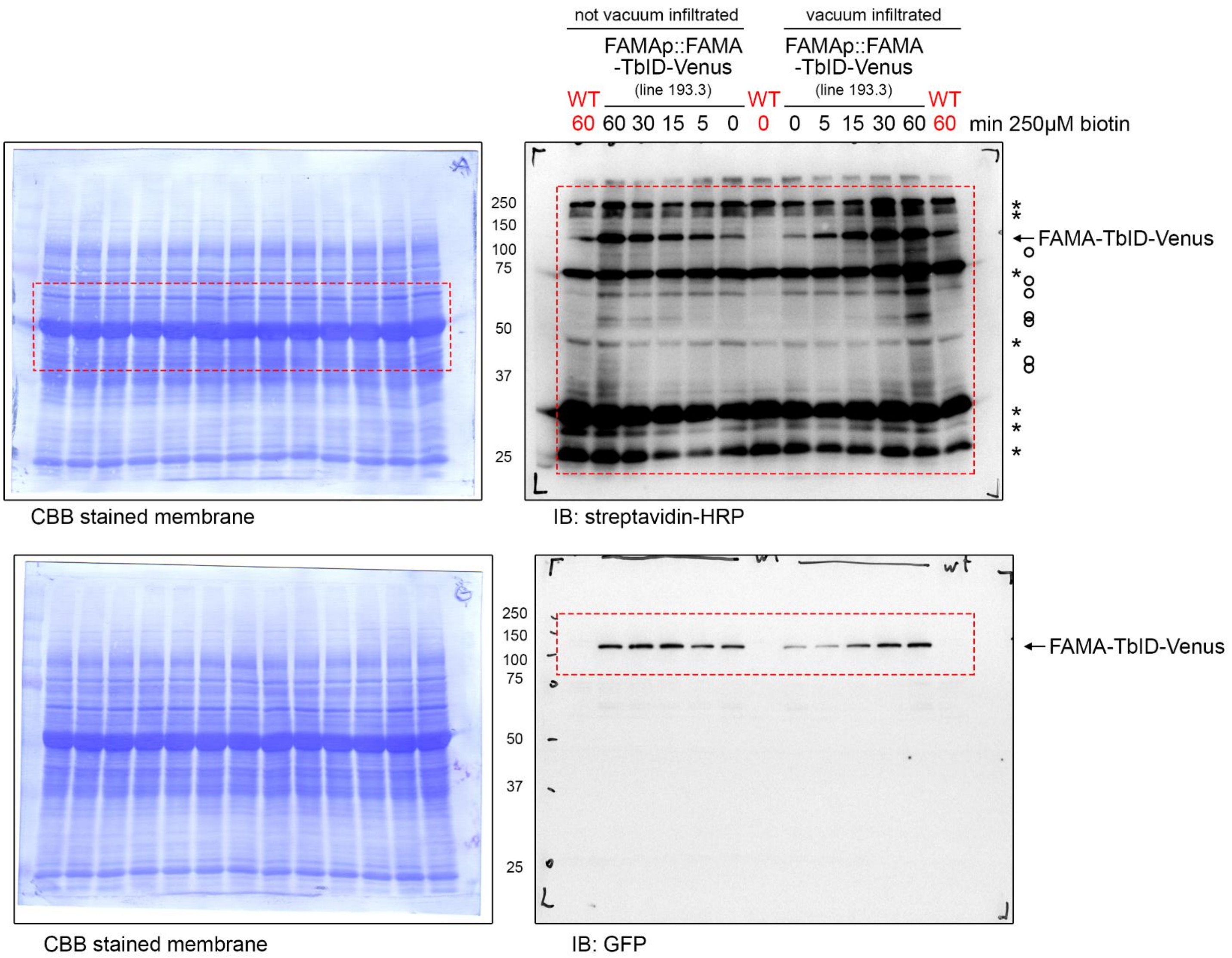

Figure 4 – figure supplement 4

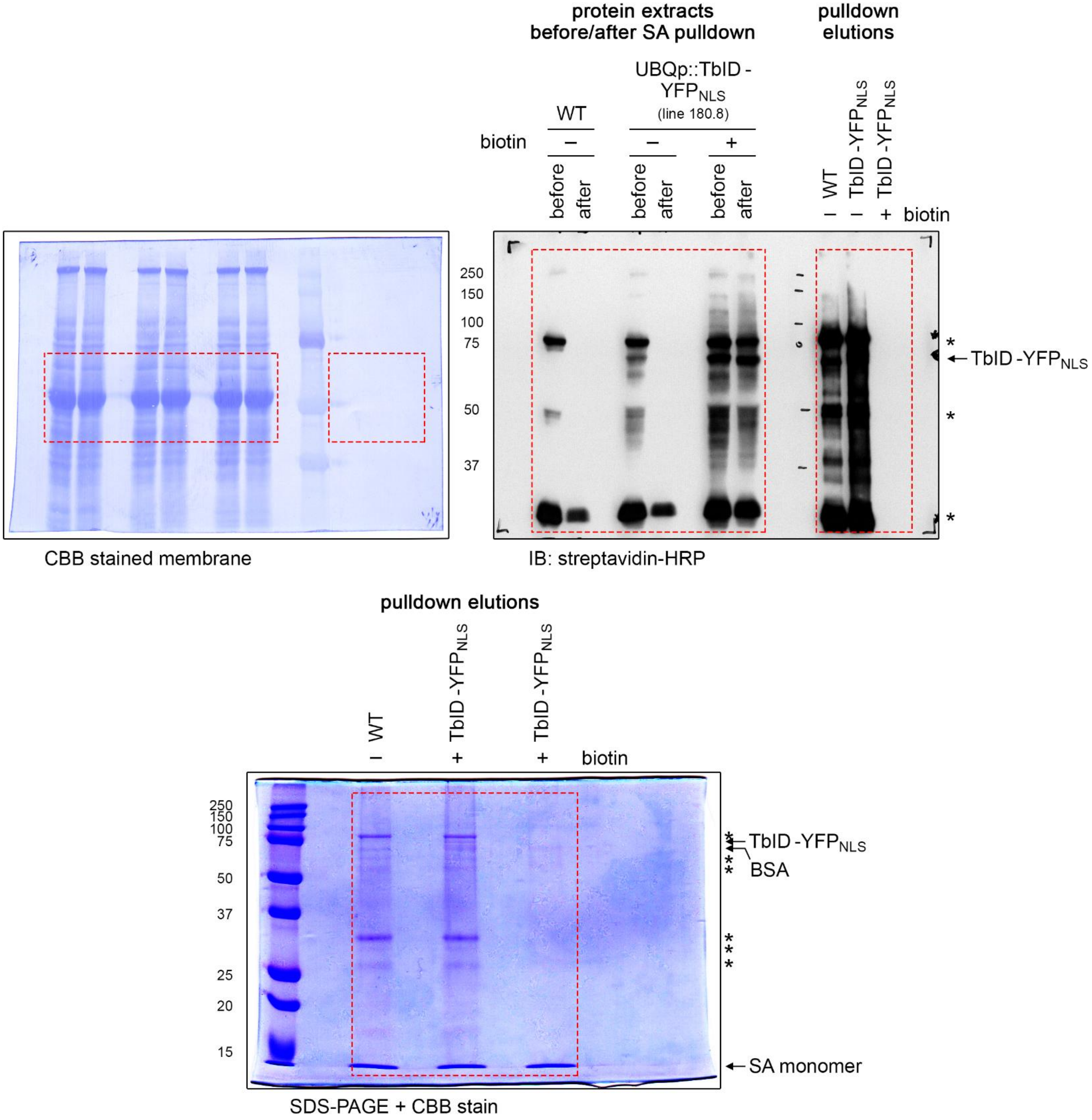

Figure 4 – figure supplement 5

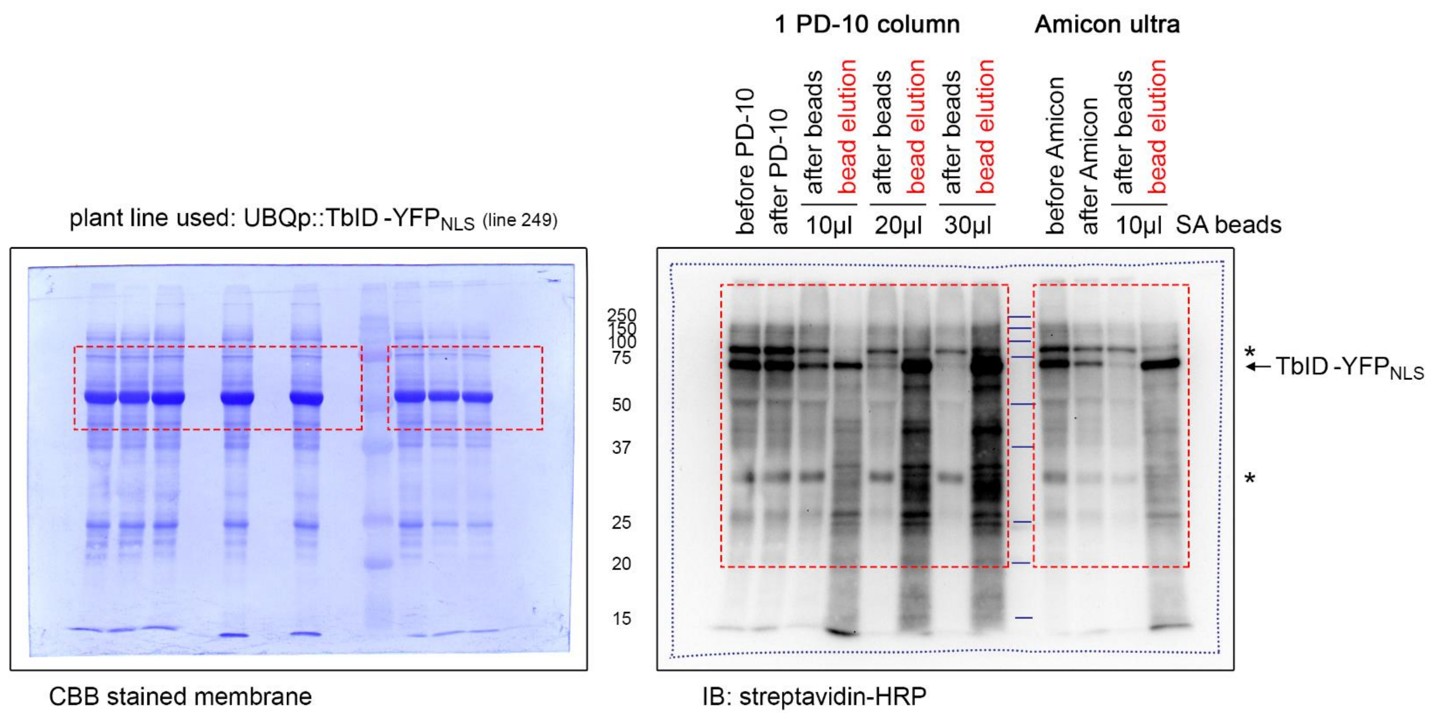

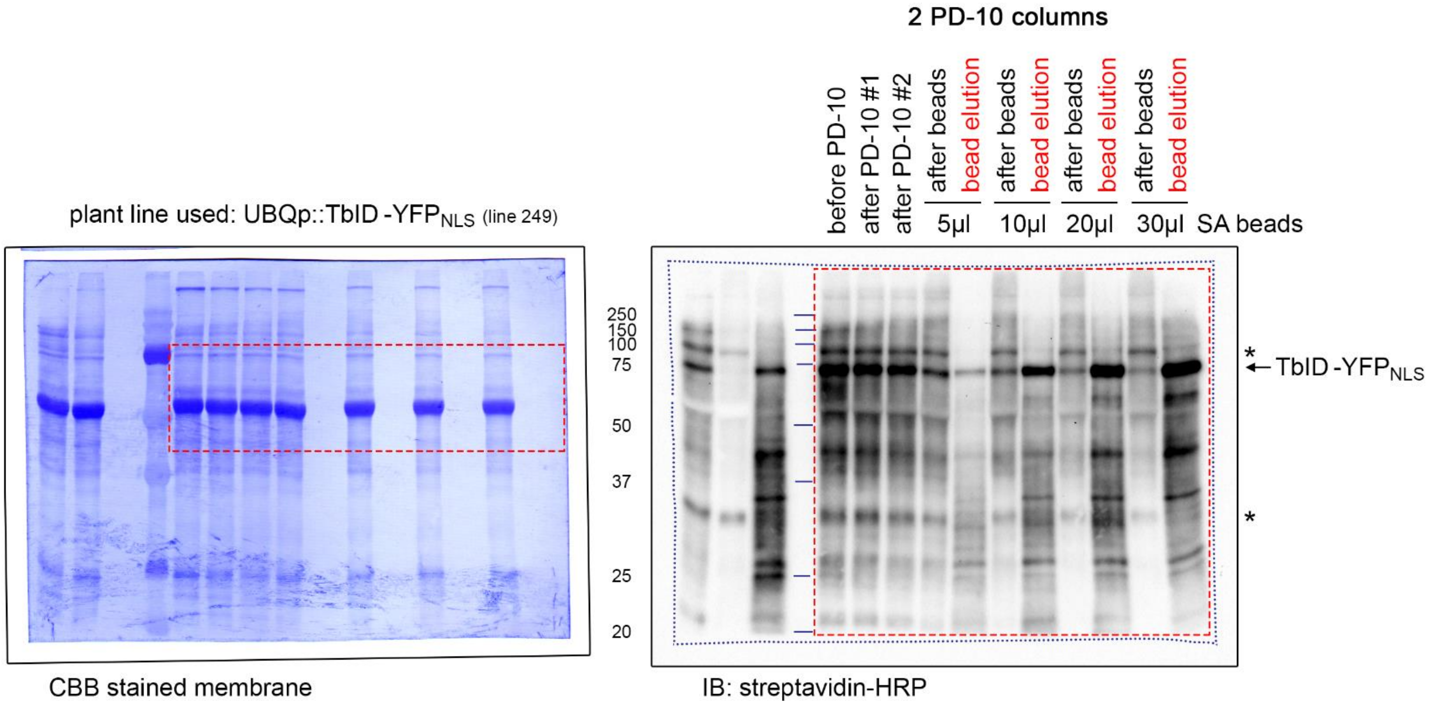

Figure 4 – figure supplement 6

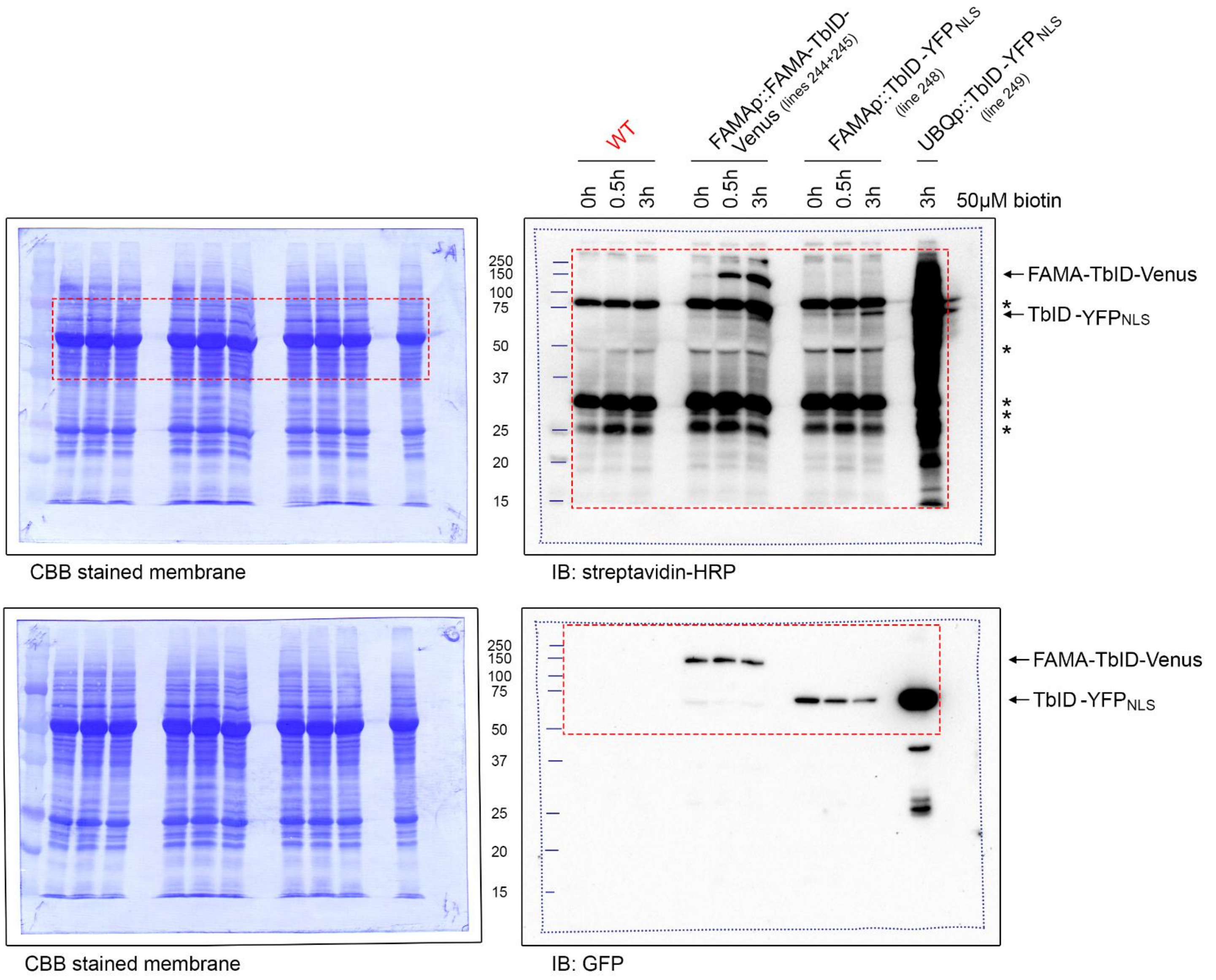

Figure 4 – figure supplement 7

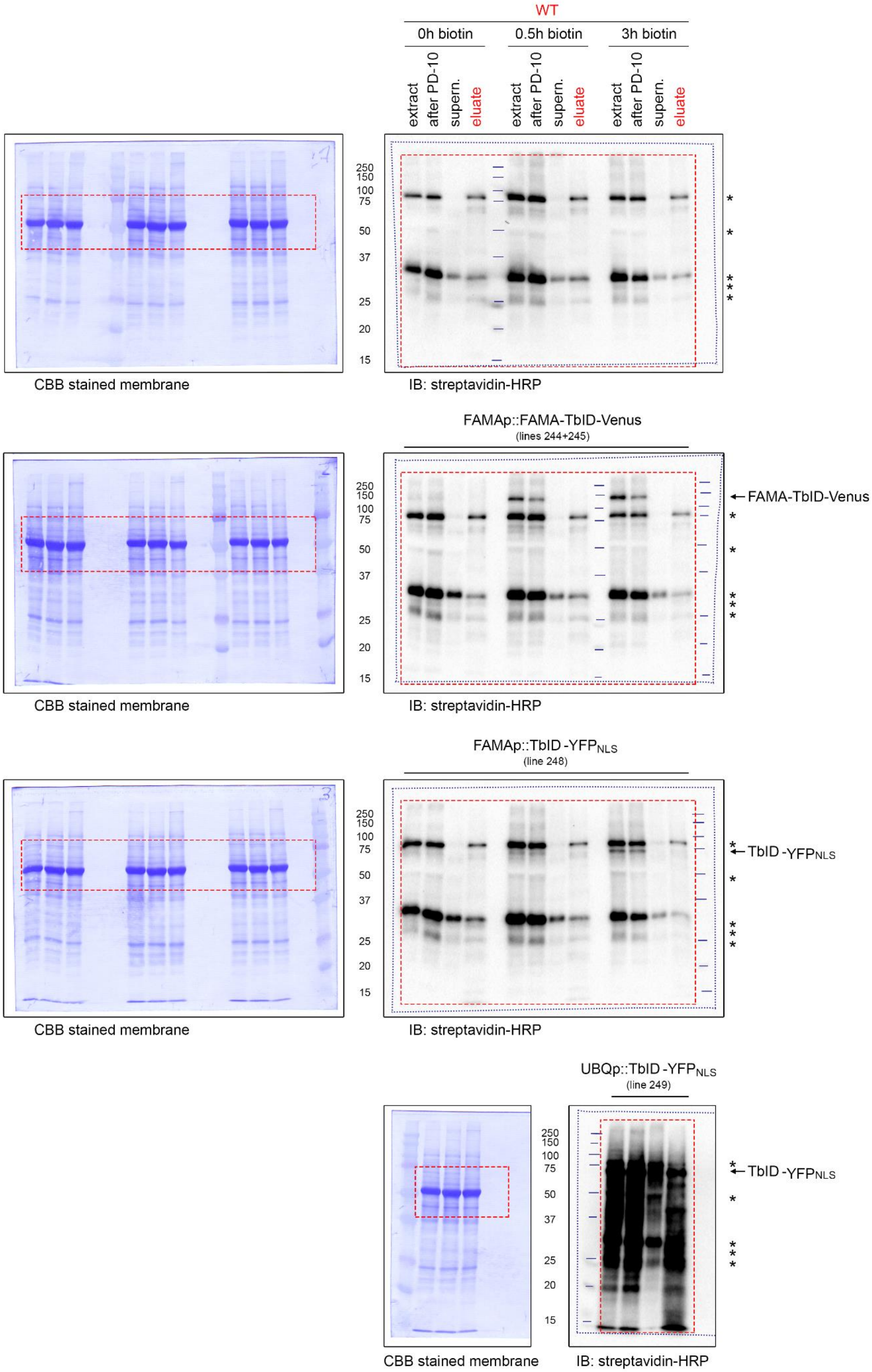

Figure 5 – figure supplement 5

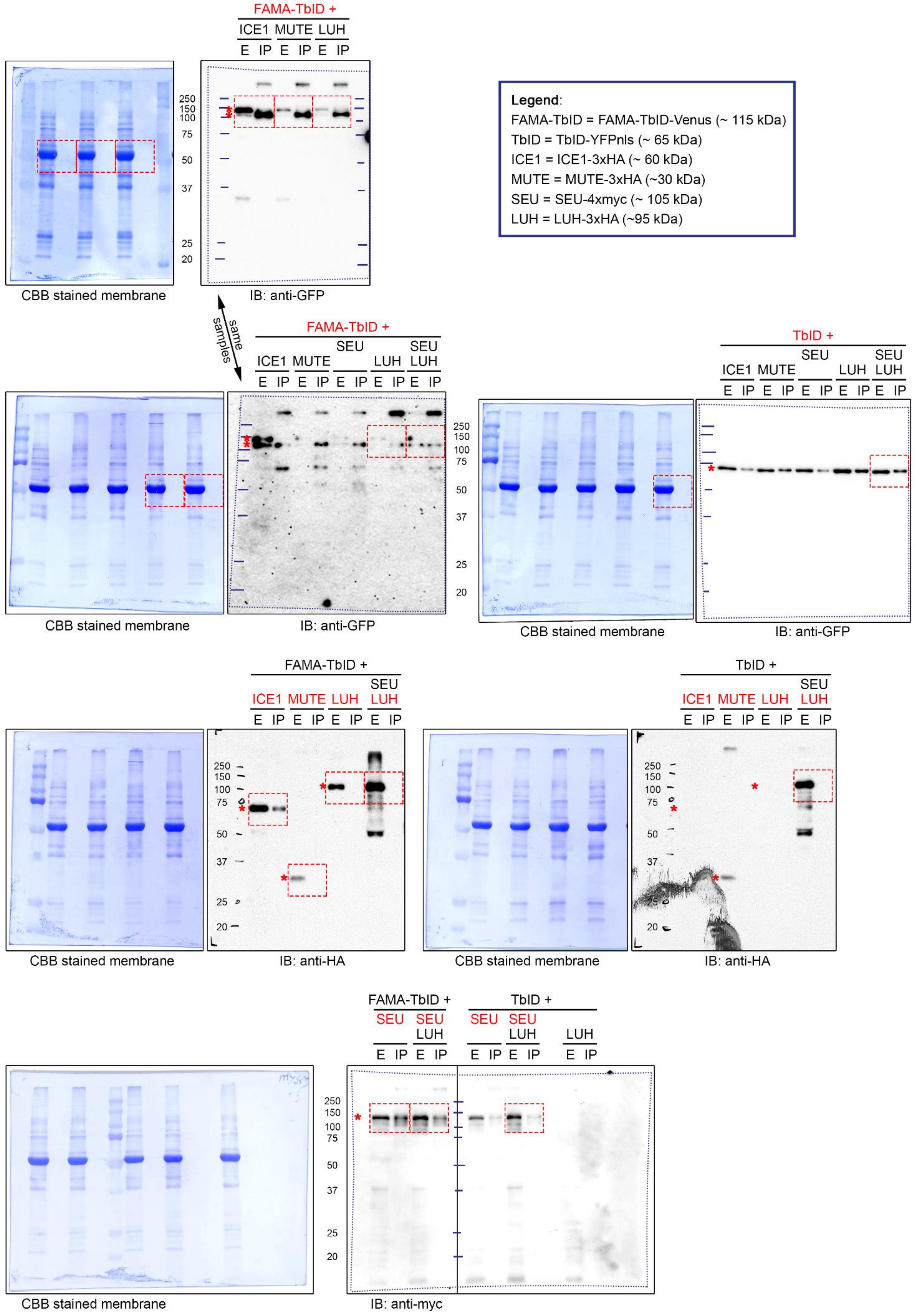

Supplement: Supplementary file 1. [file elife-47864-supp1.pdf]
