# Supplementary material for: Proximity labeling of protein complexes and cell-type-specific organellar proteomes in Arabidopsis enabled by TurboID
Source: eLife. 2019 Sep 19;8:e47864. doi: 10.7554/eLife.47864 (PMC6791687; doi:10.7554/eLife.47864)
Supplement: Transparent reporting form [file elife-47864-transrepform.docx]

***eLife’s* transparent reporting form**

We encourage authors to provide detailed information *within their submission* to facilitate the interpretation and replication of experiments. Authors can upload supporting documentation to indicate the use of appropriate reporting guidelines for health-related research (see [EQUATOR Network](http://www.equator-network.org/%20)), life science research (see the [BioSharing Information Resource](https://biosharing.org/)), or the [ARRIVE guidelines](http://www.plosbiology.org/article/info:doi/10.1371/journal.pbio.1000412) for reporting work involving animal research. Where applicable, authors should refer to any relevant reporting standards documents in this form.

If you have any questions, please consult our Journal Policies and/or contact us: [editorial@elifesciences.org](mailto:editorial@elifesciences.org).

**Sample-size estimation**

- You should state whether an appropriate sample size was computed when the study was being designed
- You should state the statistical method of sample size computation and any required assumptions
- If no explicit power analysis was used, you should describe how you decided what sample (replicate) size (number) to use
- Information on sample-size decision can be found in the material and methods section of the PL experiments and the AP-MS experiments (‘‘FAMA interactome’ and ‘nuclear proteome’ PL experiments – Seedling growth and biotin treatment’ and ‘FAMA-CFP AP-MS experiments – Affinity purification ‘).

Please outline where this information can be found within the submission (e.g., sections or figure legends), or explain why this information doesn’t apply to your submission:

**Replicates**

- You should report how often each experiment was performed
- You should include a definition of biological versus technical replication
- The data obtained should be provided and sufficient information should be provided to indicate the number of independent biological and/or technical replicates
- If you encountered any outliers, you should describe how these were handled
- Criteria for exclusion/inclusion of data should be clearly stated
- High-throughput sequence data should be uploaded before submission, with a private link for reviewers provided (these are available from both GEO and ArrayExpress)

Please outline where this information can be found within the submission (e.g., sections or figure legends), or explain why this information doesn’t apply to your submission:

- For Immunoblots shown in Figures 1-4 and the corresponding supplemental figures, information on sample composition (pool size) can be found in the figure legends and information on the number of times an experiment was done can be found in the materials and methods section describing biotin activity assays in *N. benthamiana* and Arabidopsis (‘Transformation and biotin activity assays in *N. benthamiana*‘ and ‘Plant growth conditions and biotin assays in Arabidopsis‘).
- For optimization of affinity purification of biotinylated proteins (pulldowns without and with biotin depletion), sample description and number of experiments performed are described in the materials and methods section on optimization of SA pulldown conditions (‘Optimization of streptavidin (SA) pulldown conditions – Saturation of SA beads by free biotin‘ and ‘ – Testing of biotin depletion strategies and optimization of the SA bead amount‘).
- For proximity labeling and affinity purification mass spectrometry (MS) experiments, information on the sample composition and number of replicates can be found in the materials and methods section (‘‘FAMA interactome’ and ‘nuclear proteome’ PL experiments – Seedling growth and biotin treatment‘), in the figure or figure legends of Figures 4-6 and their supplements and in the results section (‘Testing TurboID’s potential to identify partners of a very low-abundant TF and to explore the nuclear proteome of a rare and transient cell type‘). Biological replicates are described in the materials and methods section (‘‘FAMA interactome’ and ‘nuclear proteome’ PL experiments – Seedling growth and biotin treatment‘).
- For analysis of MS data, filtering of identified proteins (exclusion of contaminants and ‘low confidence’ identifications) is described in the materials and methods sections on MS data analysis (‘‘FAMA interactome’ and ‘nuclear proteome’ PL experiments – MS data analysis – identification of enriched proteins (plus sub-headings)‘ and ‘FAMA-CFP AP-MS experiments – MS data analysis‘).
- Raw data obtained from MS experiments have been deposited to the ProteomeXchange Consortium (http://proteomecentral.proteomexchange.org) via the PRIDE partner repository. Reviewer account information for access to the data are provided in the materials and methods section (‘‘FAMA interactome’ and ‘nuclear proteome’ PL experiments – MS data analysis – protein identification and label-free quantification‘ and ‘FAMA-CFP AP-MS experiments – MS data analysis‘). Tables containing identified proteins and additional information such as fold change and results of statistical analysis are provided as source files.

**Statistical reporting**

- Statistical analysis methods should be described and justified
- Raw data should be presented in figures whenever informative to do so (typically when N per group is less than 10)
- For each experiment, you should identify the statistical tests used, exact values of N, definitions of center, methods of multiple test correction, and dispersion and precision measures (e.g., mean, median, SD, SEM, confidence intervals; and, for the major substantive results, a measure of effect size (e.g., Pearson's r, Cohen's d)
- Report exact p-values wherever possible alongside the summary statistics and 95% confidence intervals. These should be reported for all key questions and not only when the p-value is less than 0.05.
- Statistical tests and multiple sample corrections used are described in brief in the figure legends and in more detail in the materials and methods section on MS data analysis (‘‘FAMA interactome’ and ‘nuclear proteome’ PL experiments – MS data analysis – identification of enriched proteins (plus sub-headings)‘ and ‘FAMA-CFP AP-MS experiments – MS data analysis‘).
- Values of N for comparisons (number of proteins used for each statistical test) can be found in the source data tables for Figure 5 and 6 and in the figure supplements for figures 5 and 6.
- Fold-changes, p- and q-values from comparisons and significantly enriched proteins can be found in the source data tables for Figure 5 and 6.

Please outline where this information can be found within the submission (e.g., sections or figure legends), or explain why this information doesn’t apply to your submission:

(For large datasets, or papers with a very large number of statistical tests, you may upload a single table file with tests, Ns, etc., with reference to sections in the manuscript.)

**Group allocation**

- Indicate how samples were allocated into experimental groups (in the case of clinical studies, please specify allocation to treatment method); if randomization was used, please also state if restricted randomization was applied
- Indicate if masking was used during group allocation, data collection and/or data analysis
- Information on sample allocation can be found in Figures 4-6, the results section (‘Testing TurboID’s potential to identify partners of a very low-abundant TF and to explore the nuclear proteome of a rare and transient cell type’, ‘Proximity labeling is superior to AP-MS for identification of candidate interactors of FAMA’ and ‘Proximity labeling can be used to analyze the nuclear proteome in rare FAMA-expressing cells during GC development’) and the materials and methods section (‘‘FAMA interactome’ and ‘nuclear proteome’ PL experiments - MS data analysis – identification of enriched proteins (and subheadings)’ and ‘FAMA-CFP AP-MS experiments – Seedling growth and crosslinking and MS data analysis’).

Please outline where this information can be found within the submission (e.g., sections or figure legends), or explain why this information doesn’t apply to your submission:

**Additional data files (“source data”)**

- We encourage you to upload relevant additional data files, such as numerical data that are represented as a graph in a figure, or as a summary table
- Where provided, these should be in the most useful format, and they can be uploaded as “Source data” files linked to a main figure or table
- Include model definition files including the full list of parameters used
- Include code used for data analysis (e.g., R, MatLab)
- Avoid stating that data files are “available upon request”
- Source data and/or additional data tables listing enriched proteins with results from the statistical analysis are provided for Figure 5 (and supplements 1, 3 and 4) and Figure 6 (and supplements 2 and 3).

Please indicate the figures or tables for which source data files have been provided:
